# Single‐cell RNA sequencing infers the role of malignant cells in drug‐resistant multiple myeloma

**DOI:** 10.1002/ctm2.653

**Published:** 2021-12-17

**Authors:** He‐nan Wang, Jing Yang, De‐Huan Xie, Zhigang Liang, Yang Wang, Rui‐ying Fu, Xindi Liu, Zhong‐jun Xia, Guangshuai Jia, Liang Wang

**Affiliations:** ^1^ Department of Hematology, Beijing Tongren Hospital Capital Medical University Beijing China; ^2^ Department of Nasopharyngeal Carcinoma, State Key Laboratory of Oncology in South China Sun Yat‐sen University Cancer Center Guangzhou China; ^3^ State Key Laboratory of Respiratory Disease Affiliated Cancer Hospital & Institute of Guangzhou Medical University Guangzhou China; ^4^ Beijing Key Laboratory of Nasal Diseases Beijing Institute of Otorinolaringology Beijing China; ^5^ Department of Hematologic Oncology Sun Yat‐sen University Cancer Center Guangzhou China; ^6^ Beijing Advanced Innovation Center for Big Data‐Based Precision Medicine Beihang University & Capital Medical University, Beijing Tongren Hospital Beijing China

Dear Editor,

Using single‐cell RNA‐sequencing (scRNA‐seq), we identified special populations that might be involved in the progression of drug resistance and various poor prognostic biomarkers in multiple myeloma (MM).

Although the overall treatment outcomes of MM have been improved,^1^ challenges still exist in relapsed MM due to the lack of effective drugs and predictive biomarkers.^2^, ^3^ ScRNA‐seq has been applied to unbiasedly identify the cellular heterogeneity and novel biomarkers.^4^, ^5^ To unravel the tumour microenvironment dynamics associated with carcinogenesis and survey cellular heterogeneity in MM, we performed scRNA‐seq on bone barrow cells from 3 primary (newly diagnosed), 1 recurrent, 3 drug‐resistant MM and 1 healthy donor (Figure S1A and C; Table S1; Supplementary materials). After rigorous quality control (Figure S1B; Supplementary materials), 52 793 cells were obtained for further analysis based on known marker genes and differentially expressed genes (DEGs) (Table S2). Notably, we retrieved B cells, T cells, NK cells, myeloid cells, DCs, erythrocytes, haematopoietic progenitor cells (HPCs) (Figure 1A) and key marker genes in these clusters, that is *NKG7* for NK cells, *CD34* for HPCs and *MZB1* for B cells (Figure 1B). Further, we found B, T and myeloid cells were highly enriched in drug‐resistant MM (Figure 1C and E), suggesting that B cells, T cells and myeloid cells might be involved in the progression of drug resistance in MM. Our results showed substantial variation in B cells proportion in MM compared with control group (Figure 1D).

**FIGURE 1:**
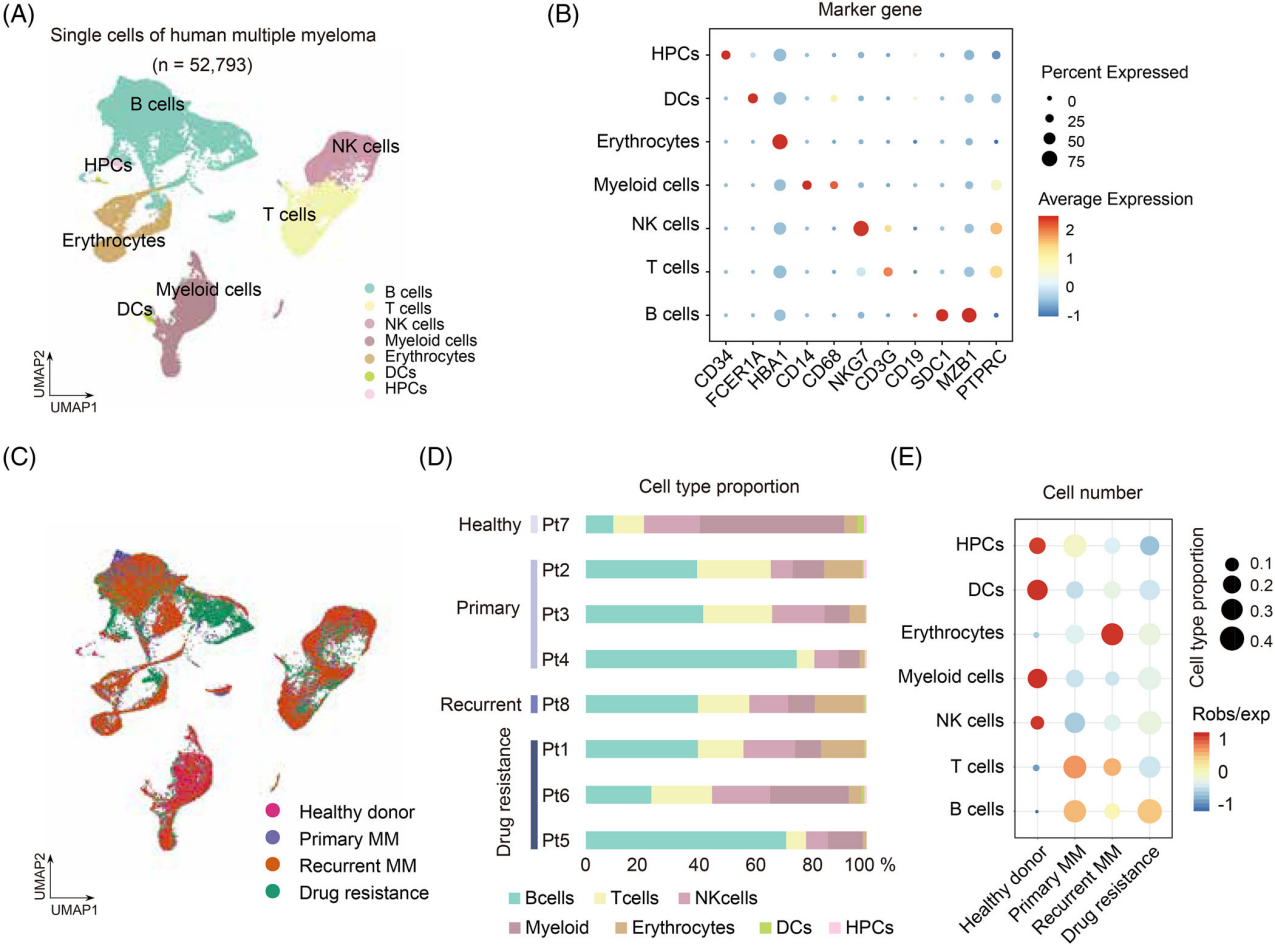
Single‐cell transcriptional profiles in multiple myeloma (MM). (A) Identification of cellular types using single cells of human multiple myeloma. (B) Dot plot showing the canonical marker genes in 7 cellular types. (C) UMAP plot showing the distribution of cells in different conditions including control group, primary MM, recurrent MM and drug‐resistant MM. (D) Cell type proportion of all patients with or without MM. (E) Cell type proportion of cellular types in different conditions including control group, primary MM, recurrent MM and drug‐resistant MM. UMAP: Uniform Manifold Approximation and Projection; Pt: patients

We then focus on B cells, T cells and myeloid cells using unsupervised clustering and Uniform Manifold Approximation and Projection (UMAP).^6^ First, B cells were clustered into 12 clusters or four subgroups according to gene expression of *MS4A1, CD19, SDC1* and *MKI67* (Figure 2A and B; Table S3). Clusters 1, 3, 5 and 7 were predominantly enriched in drug‐resistant group, and cluster 1 and 7 are proliferating/cycling cells with high expression of *MKI67* (Figure 2B and C), suggesting that B cells proliferation may contribute to the progression of drug resistance of MM. Besides, we calculated the large‐scale chromosomal copy number variations (CNV) to distinguish the malignant B cells from normal cells.^7^ Drug‐resistant MM showed remarkably highest CNV levels among groups (Figure 2D). Interestingly, cluster 5 malignant B cells that is significantly enriched in drug‐resistant MM (*p* < .05, *χ* ^2^ test) (Figure 2C) exhibited high CNV levels (Figure 2D and E), suggesting that cluster 5 B malignant cells were the major source of malignant cells in drug resistance. Further, we focused on cluster 5 and identified DEGs through the comparison of drug‐resistant MM versus primary MM (Figure 2F; Table S4) and found several marker genes in this subpopulation including CD27. By ordering these cells to reconstruct pseudo‐time trajectories,^8^ we observed cluster 5 cells bifurcated to 2 branches, the drug‐resistant MM and the relapsed MM, suggesting distinct cellular differentiation paths of these two MM stages (Figure 2G). Importantly, we identified novel genes of *CCL4, TNFRSF17, LMAN2* and *MZB1* that were positively correlated with drug resistance (Figure 2H). Concordantly, the patients with higher expression of these genes [i.e., *CCL4* (*p* = .017), *TNFRSF17* (*p* = .0082), *LMAN2* (*p* = .025) and *MZB1* (*p* = .016)] had poorer prognosis than those with low expression (Figure 2I). This data further supports that cluster 5 cells expressing *CCL4, TNFRSF17, LMAN2* and *MZB1* contribute to drug resistance.

**FIGURE 2:**
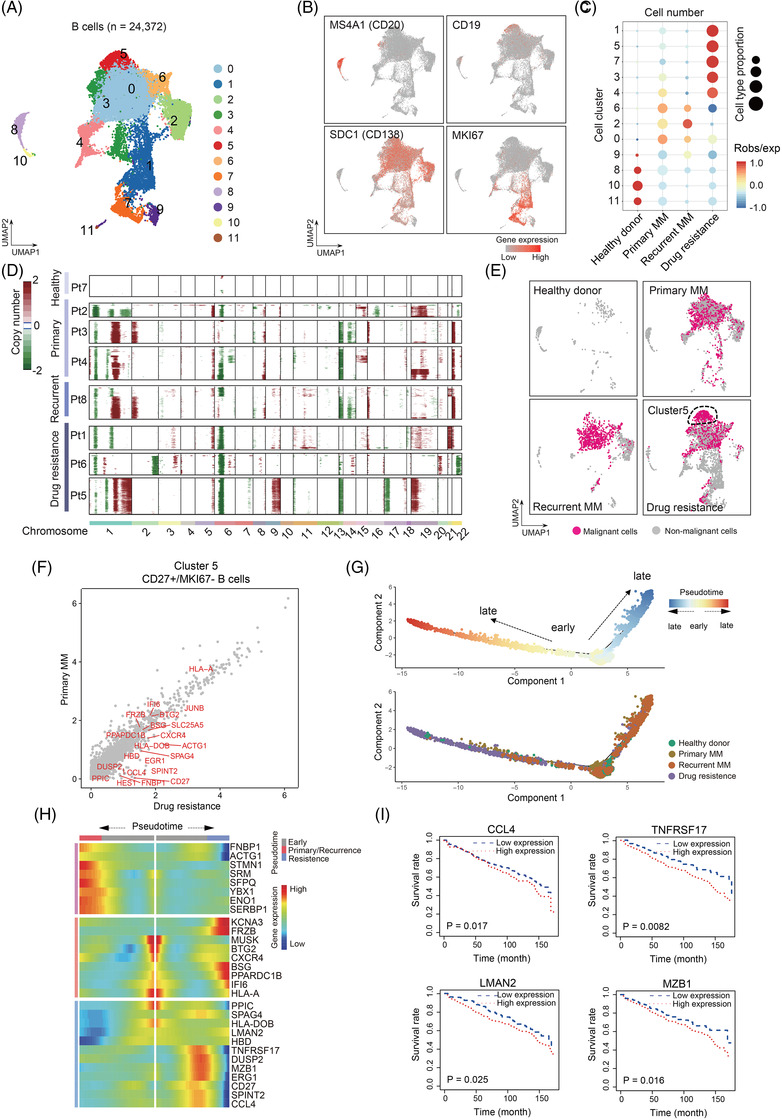
Transcriptional landscape of B cells in MM. (A) UMAP plot suggesting identification of cellular subtypes in B cells. (B) Scatterplot showing expression of marker genes in B cells; (C) Cell type proportion of cellular subtypes in B cells in different conditions including control group, primary MM, recurrent MM and drug‐resistant MM. (D) Heat map showing large‐scale CNVs of B cells from 8 patients. The red colour represents high CNV level and blue represents low CNV level. (E) The distribution of malignant cells in different conditions including control group, primary MM, recurrent MM and drug‐resistant MM. (F) Differentially expressed gene profiles along malignant progression. (G) Pseudo‐time of malignant B cells with different conditions. (H) The genes along the pseudo‐time were clustered hierarchically into three profiles, including early, primary/recurrence and resistance. (I) The Kaplan‐Meier survival analysis in MM patients based on the expression of CCL4, TNFRSF17, LMAN2 and MZB1. CNV: chromosomal copy number variations

Next, we subset a total of 8371 T cells and partitioned them into 14 clusters or two distinct clusters of canonical CD8 and CD4 T cells (Figure 3A and B). We then examined canonical T cell genes and DEGs for each subpopulation (Figure 3C; Table S5). We observed that clusters 1, 2, 5 and 10 were CD8+ T cells dominated in drug‐resistant MM (Figure 3D), indicating that CD8+ T cells were involved in the development of drug resistance. Moreover, the proportion of cluster 10 CD8+ T cells was higher in drug‐resistant MM patients, yet presented a compromised cytotoxic function and an exhausted state with higher proliferation ability, suggesting that such cells lose anti‐tumour function (Figure 3E). Further, we performed deconvolution analysis on TCGA MM dataset, and found that CD8+ T cells in relapsed and resistance MM were in activated cytotoxic and exhausted state with higher proliferation ability comparing with primary ones (Figure S1D).

**FIGURE 3:**
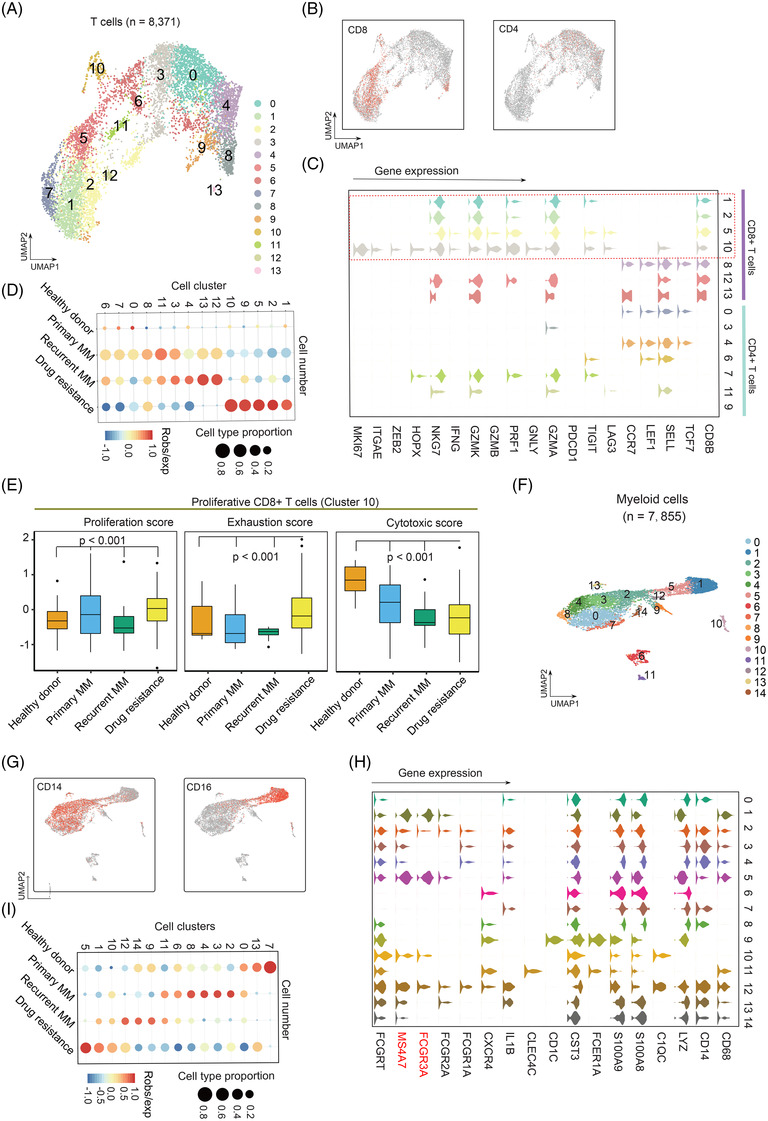
Transcriptional landscape of T cells and myeloid cells in MM. (A) UMAP plot suggesting identification of cellular subtypes in T cells. (B) Scatterplot showing expression of marker genes in CD4+ and CD8+ T cells; (C) Violin plot showing the canonical marker genes in CD4+ T cells and CD8+ T cells. (D) Cell type proportion of cellular subtypes in T cells in different conditions including control group, primary MM, recurrent MM and drug‐resistant MM. (E) The striking accumulation of CD8+ T cells with lowly cytotoxic and highly proliferative states in drug resistance. (F) UMAP plot suggesting identification of cellular subtypes in myeloid cells. (G) Scatterplot showing expression of marker genes in myeloid cells including CD14+ and CD16+. (H) Violin plot showing the canonical marker genes in CD14+ and CD16+ myeloid cells. (I) Cell type proportion of cellular subtypes in myeloid cells in different conditions including control group, primary MM, recurrent MM and drug‐resistant MM

Lastly, we subset 7855 myeloid cells and partitioned them into 15 clusters or two subsets of CD14 and CD16 myeloid cells (Figure 3F and G; Table S6). Clusters 1, 5, 10 and 12 were classified as CD16+ and the others were CD14+ myeloid cell according to the known marker genes (Figure 3G and H). Notably, *FCGR3A* and *MS4A7* were highly expressed in CD16+ myeloid cells, indicating their potential activity towards MM cells when treated with monoclonal antibodies.^9^ Clusters 1, 5, 10 and 12 were dramatically enriched in drug‐resistance group (Figure 3I), suggesting that CD16+ myeloid cells involved in development of drug‐resistant MM. Concordantly, CD16+ cells showed higher distribution in in relapsed/resistance MM comparing with primary samples (Figure S1E).

Cell–cell interactions analysis by CellphoneDB^10^ revealed that B cells and myeloid cells in drug‐resistant patients had higher correlation and interactions with other cells (Figure 4A). Furthermore, cluster 5 malignant B cells had a positive correlation with CD16+ myeloid cells (cluster 5 myeloid cells) and a negative correlation with CD14+ myeloid cells (cluster 7 myeloid cells) in primary MM cells (Figure 4B). Interestingly, CD8+ T cells (cluster 10 T cells) correlated most with CD16+ myeloid cells in drug‐resistant MM cells (Figure 4C). Furthermore, ligand–receptor interactions analysis revealed that LAMP1–FAM3C, CCL4–GPRC5D and CCL4–SLC7A1 pairs might mediate the interaction between malignant B cells and CD16+ myeloid cells in primary MM (Figure 4D), while CD74–COPA, CD74–MIF and CCL4–CCR5 may contribute to the progression of drug‐resistant MM between CD8+ T cells and CD16+ myeloid cells in drug‐resistant MM cells (Figure 4E).

**FIGURE 4:**
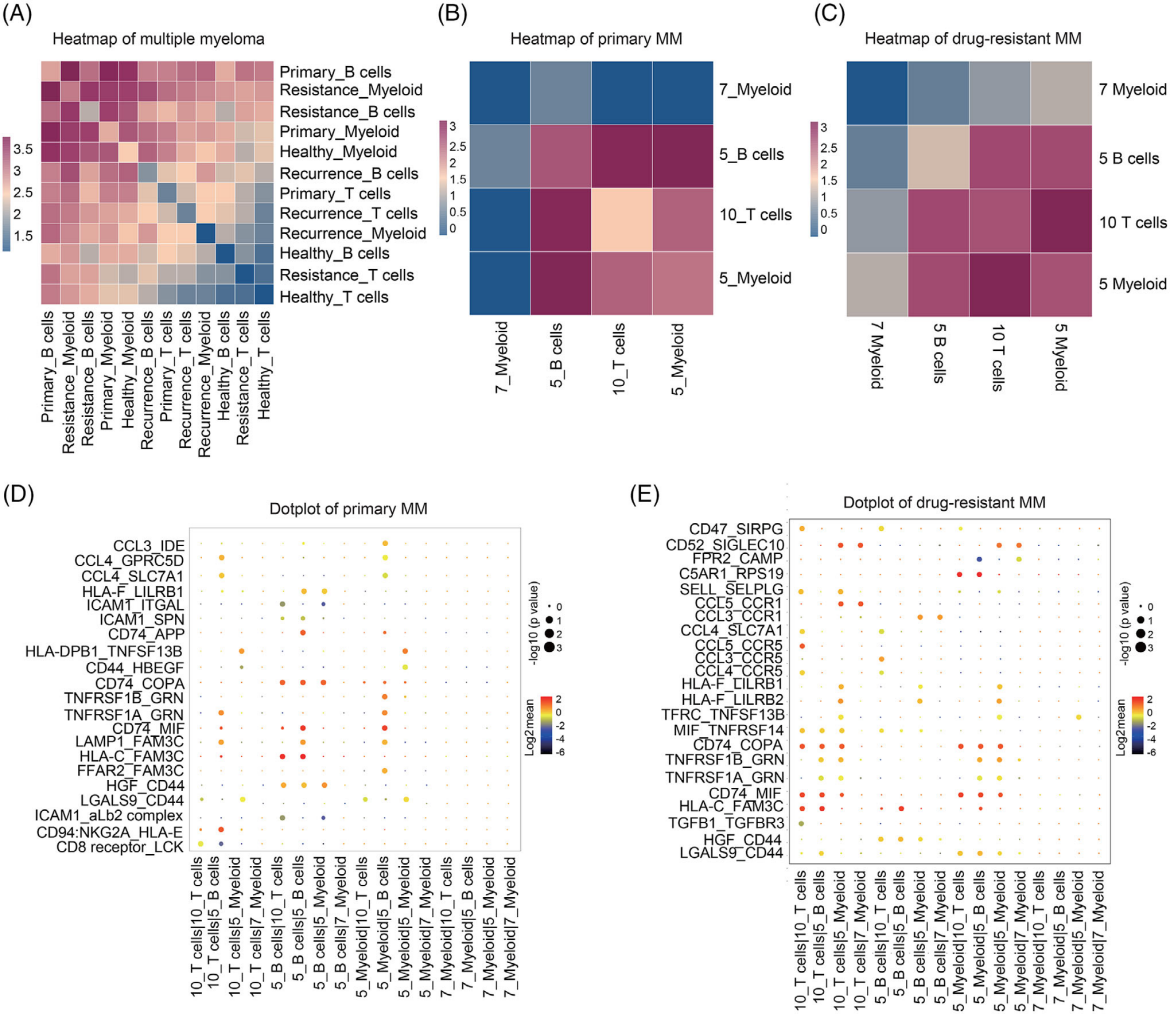
Interaction between innate immune cells and adaptive immune cells in MM. (A) Heat map showing the interaction in different conditions among immune cells including T cells, B cells and myeloid cells. (B) Interaction among malignant B cells, CD8+ T cells, CD14+ myeloid cells and CD16+ myeloid cells in primary MM. (C) Interaction among malignant B cells, CD8+ T cells, CD14+ myeloid cells and CD16+ myeloid cells in drug‐resistant MM. (D) Dot plot showing the interaction between ligands and receptors among immune cells in primary MM. (E) Dot plot showing the interaction between ligands and receptors among immune cells in drug‐resistant MM

In conclusion, we identified a malignant B cell subpopulation that is specifically enriched in drug‐resistant MM. The exhausted CD8+ T and distinct CD16+ myeloid cells were associated with the progression of drug‐resistant MM. Our data provide biological insights into novel therapeutic targets and biomarkers for drug‐resistant MM.

## FUNDING

This work was financially supported through grants from the National Natural Science Foundation of China (81873450, 82170181 to L.W. and 31900570 to G.J.), and the Open Research Fund from Beijing Advanced Innovation Center for Big Data‐Based Precision Medicine, Beijing Tongren Hospital, Beihang University & Capital Medical University (grant No. BHTR‐KFJJ‐202009) to L.W.

## CONFLICT OF INTEREST

All the authors declare no interest of conflicts.

## Supporting information

Figure S1

Supporting Information

Table S1

Table S2

Table S3

Table S4

Table S5

Table S6
