# Supplementary figures and images for: Single‐cell RNA sequencing infers the role of malignant cells in drug‐resistant multiple myeloma
Source: Clin Transl Med. 2021 Dec 17;11(12):e653. doi: 10.1002/ctm2.653 (PMC8678945; doi:10.1002/ctm2.653)

Figure S1

A

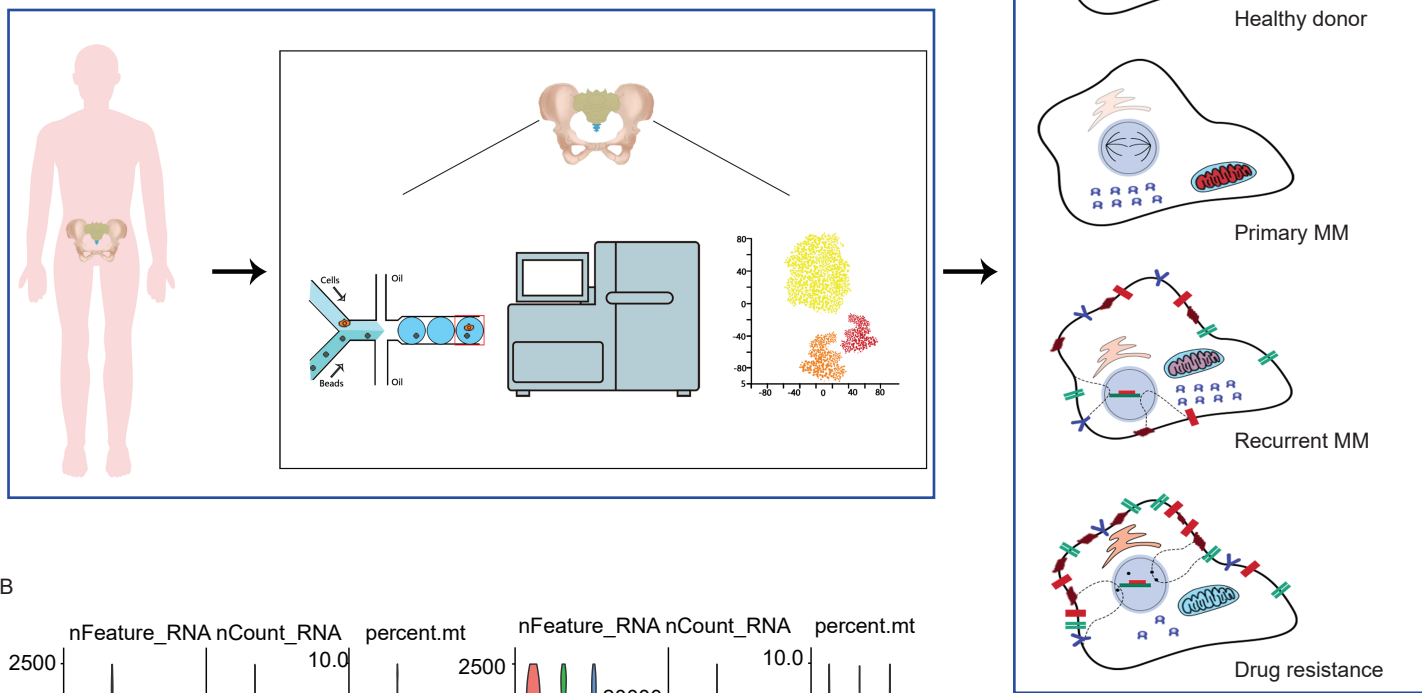

B

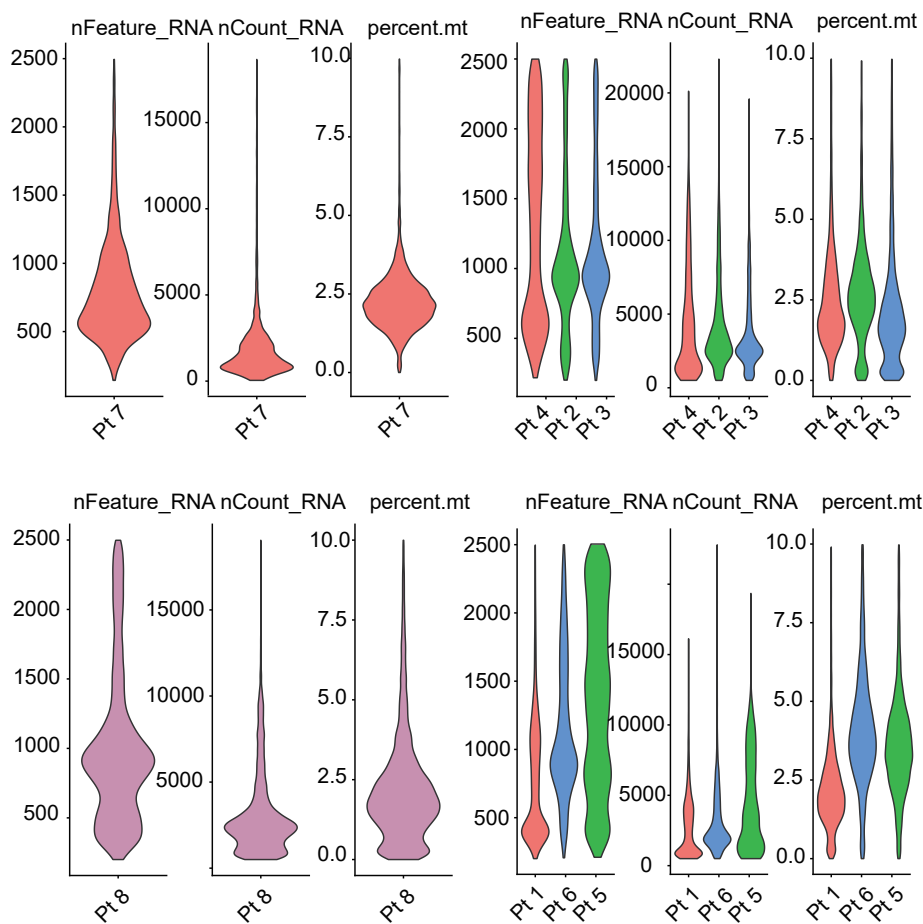

C

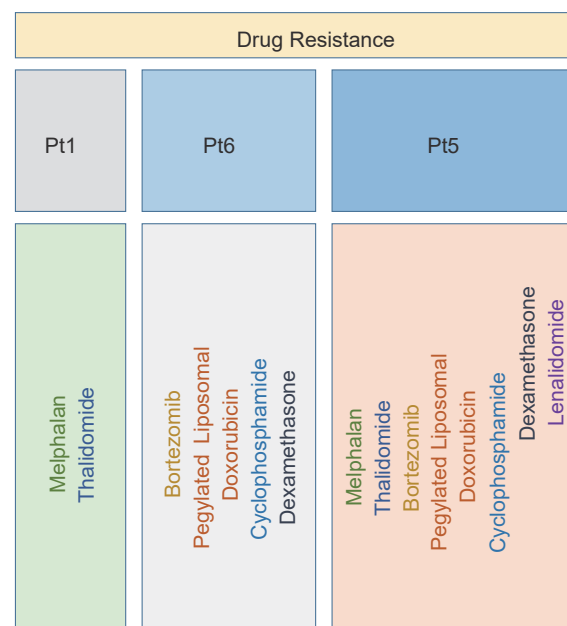

D

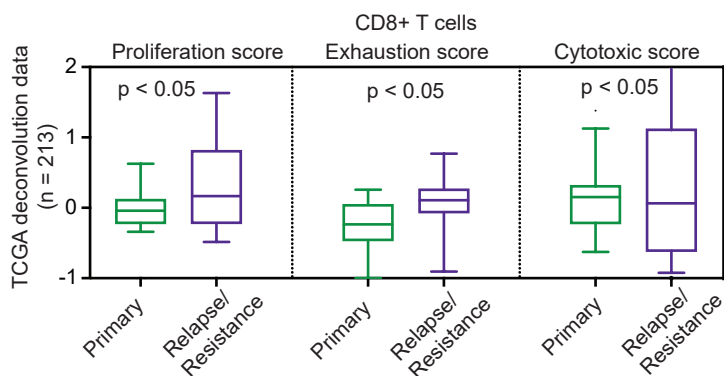

E

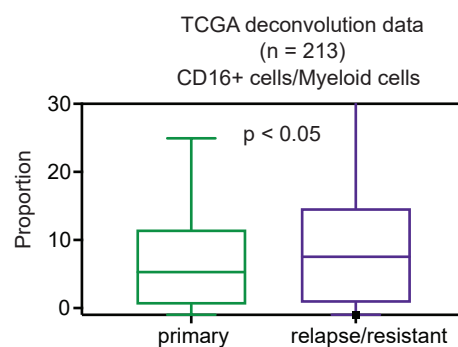

Supplement: Supplementary file 1 — Figure S1 [file CTM2-11-e653-s008.pdf]
