# Supplementary material for: Single‐cell RNA sequencing infers the role of malignant cells in drug‐resistant multiple myeloma
Source: Clin Transl Med. 2021 Dec 17;11(12):e653. doi: 10.1002/ctm2.653 (PMC8678945; doi:10.1002/ctm2.653)
Supplement: Supplementary file 2 — Supporting Information [file CTM2-11-e653-s002.docx]

**Supplementary Materials**

**Methods**

**MM samples**

We obtained the 8 human fresh bone marrow samples including 1 patient with normal bone marrow (this patient was previously diagnosed as primary central nervous lymphoma without bone marrow infiltration, and achieved complete remission at the time of bone marrow sampling, which was used as the control group), 3 patients with primary MM (patient 2, patient 3 and patient 4), 1 patient (patient 8) with recurrent MM and 3 patients with drug-resistant MM (patient 1, patient 5 and patient 6). Patient 1 was resistant to thalidomide and melphalan. Patient 5 received bortezomib, pegylated liposomal doxorubicin, cyclophosphamide, and dexamethasone, but failed to respond. Patient 6 received lenalidomide, bortezomib, pegylated liposomal doxorubicin, cyclophosphamide, and dexamethasone, but relapsed after all these regimens. All the patients signed the informed consent. The present study was approved by the institutional review board of Beijing Tongren Hospital and Sun Yat-sen University Cancer Center. The clinical features were presented in Supplementary Table 1.

**Single-cell RNA sequencing**

As described previously, scRNA-seq was used to detect transcriptionally coherent populations [1]. Briefly, single-cell 3’ Library and Gel Bead Kit V3 (10× Genomics, 1000075) and Chromium Single Cell B Chip Kit (10× Genomics, 1000074) were used to prepare the scRNA-seq cDNA libraries according to the manufacture’s protocol. Subsequently, the sequencing was performed on Illumina HiSeq X Ten with pair end 150 bp (PE150) paired-end reads.

**Processing raw data from scRNA-seq of 10× Genomics**

For raw data analysis, the fastq files were processed to generate counts of single cell using cellranger (10× Genomics, Version 3.0.2). The human genome hg19 was considered as reference genome. To obtain cells with high quality, the ratio of mitochondria lower than 0.2 and cells with genes over 2000 were maintained.

The dimension reduction and cell clustering analysis were performed by employing the Seurat (Version 3.2.2) R package. Briefly, the filtered genes were implemented a global-scaling normalization based on the method of LogNormalize by “NormalizeData” functions. The highly variable genes were calculated by “FindVariableGenes” functions. With a resolution of 0.5 as the threshold, the principal component analysis was used for cell clustering. The visualizations were shown by using urban mobility analysis platform (UMAP). Identification of differentially expressed genes was obtained by “FindAllMarkers” function using wilcox test with p < 0.05 and avg_logFC >0.25 as the cut-off criteria. The annotation of cellular types was performed using known marker genes in CellMarker databases (http://biocc.hrbmu.edu.cn/CellMarker/) and PanglaoDB database (https://panglaodb.se/) database. The proportion of cellular types were calculated using Chi-square test.Pseudotime analysis was performed using monocle2 package in R. It could help us find highly variable features and build up trajectory. In addition, the inferCNV R package was used to estimate the initial CNVs for each region, distinguishing the malignancy of cellular subtypes in B cells.

**Cell-cell communication identification**

The cell-cell communication and ligand-receptor interaction were explored by using cellphoneDB tool [2]. The cellphoneDB tool was used to predict cell-type specificity mediating with the recurrence and drug-resistance in MM.

**TCGA data analysis**

The Kaplan-Meier survival curves were performed to predict the survival rate of overall survival with the log-rank test to compare the survival curves between two groups in patients with MM based on Gene Expression Omnibus database (GSE136324). A total of 861 samples with clinical features were obtained to analyze the overall survival. The deconvolution analysis was performed using CIBERSORTx. We first retrieved the subpopulations identified by our 7 samples with 213 TCGA MM samples. Next, we used marker gene expression of the large TCGA cohort to compare the exhaustion, proliferation, and cytotoxic scores between primary and relapsed/resistance MM samples.

**Supplementary Figure Legends**

**Figure S1 Workflow depicting collection and processing of specimens of MM and control group for scRNA-seq.** (A) Procedures for acquiring transcriptional and genotypic information for scRNA-seq. (B) Data quality control of scRNA-seq using Seurat. (C) The drug information of drug-resistant MM. (D) Bar plot showing cell score in TCGA MM dataset. (E) Bar plot showing CD16+ cell proportion in total myeloid cells in TCGA MM dataset.

**Supplementary Table Legends**

**Table S1.** Clinical characteristics of multiple myeloma patients in this study.

**Table S2**. Differentially expressed genes for each cellular type in multiple myeloma.

**Table S3**. Differentially expressed genes for each cellular subtype in B cells.

**Table S4**. Differentially expressed genes in malignant (cluster 5) B cells between primary and drug-resistant MM.

**Table S5**. Differentially expressed genes for each cellular subtype in T cells.

**Table S6**. Differentially expressed genes for each cellular subtype in myeloid cells.

**Reference**

1. Mei, Y., W. Xiao, H. Hu, et al. Single-cell analyses reveal suppressive tumor microenvironment of human colorectal cancer. Clin Transl Med, 2021. 11(6): p. e422.

2. Efremova, M., M. Vento-Tormo, S.A. Teichmann, et al. CellPhoneDB: inferring cell-cell communication from combined expression of multi-subunit ligand-receptor complexes. Nat Protoc, 2020. 15(4): p. 1484-1506.
